# Supplementary material for: Placental genotype affects early postpartum maternal behaviour
Source: R Soc Open Sci. 2019 Sep 18;6(9):190732. doi: 10.1098/rsos.190732 (PMC6774950; doi:10.1098/rsos.190732)
Supplement: Supplemental File 1 [file rsos190732supp1.docx]

**Supplemental Figure 1 (Figure S1)**. Day 1 hybrid (grey) and conspecific (black) pup ultrasonic vocalizations (USVs). Hybrid pups produced significantly more USVs than conspecific pups. Bars represent mean ± 1 SE. * *p* < 0.05 (ANOVA).

| Model | dAICc | df |
| --- | --- | --- |
| null | 0.0 | 2 |
| Pregnancy Day | 1.9 | 3 |
| Pup Genotype | 2.6 | 3 |
| Pup Genotype + Pregnancy Day | 4.7 | 4 |
| Pup Genotype + Pregnancy Day + Pup Genotype:Pregnancy Day | 7.1 | 5 |

Table 1. AICc table for generalized linear models testing the effect of factors on the number of lines crossed in an open field test

| Model | dAICc | df |
| --- | --- | --- |
| null | 0.0 | 2 |
| Pup Genotype | 2.2 | 3 |
| Pregnancy Day | 2.4 | 3 |
| Pup Genotype + Pregnancy Day | 5.0 | 4 |
| Pup Genotype + Pregnancy Day + Pup Genotype:Pregnancy Day | 6.5 | 5 |

Table 2. AICc table for generalized linear models testing the effect of factors on the time spent in the center of the arena in an open field test

| Model | dAICc | df |
| --- | --- | --- |
| null | 0.0 | 2 |
| Pup Genotype | 0.3 | 3 |
| Pregnancy Day | 2.6 | 3 |
| Pup Genotype + Pregnancy Day | 3.1 | 4 |
| Pup Genotype + Pregnancy Day + Pup Genotype:Pregnancy Day | 5.9 | 5 |

Table 3. AICc table for generalized linear models testing the effect of factors on the latency to enter the center of the arena in an open field test

| Model | dAICc | df |
| --- | --- | --- |
| null | 0.0 | 2 |
| Pup Genotype | 0.5 | 3 |
| Pregnancy Day | 2.0 | 3 |
| Pup Genotype + Pregnancy Day | 2.4 | 4 |
| Pup Genotype + Pregnancy Day + Pup Genotype:Pregnancy Day | 5.3 | 5 |

Table 4. AICc table for generalized linear models testing the effect of factors on the time spent frozen at the onset of the trial in an open field test

| Model | dAICc | df |
| --- | --- | --- |
| Pup Genotype + Day Postpartum + Pup Genotype:Day Postpartum + (1\|Experience) | 0.0 | 7 |
| Pup Genotype + Day Postpartum + Pup Genotype:Day Postpartum | 1.6 | 6 |
| Pup Genotype + Day+ (1\|Experience) | 1.9 | 6 |
| Pup Genotype + (1\|Experience) | 2.1 | 5 |
| Pup Genotype + Day | 3.4 | 6 |
| Pup Genotype | 3.5 | 4 |
| null + (1\|Experience) | 3.9 | 4 |
| null | 4.7 | 3 |

Table 5. AICc table for linear mixed models testing the effect of factors on in-cage activity during the light cycle

| Model | dAICc | df |
| --- | --- | --- |
| Pup Genotype + Day Postpartum + Pup Genotype:Day Postpartum + (1\|Experience) | 0.0 | 7 |
| Pup Genotype + Day Postpartum + Pup Genotype:Day Postpartum | 0.2 | 6 |
| Pup Genotype + (1\|Experience) | 6.7 | 5 |
| Pup Genotype | 6.9 | 4 |
| Pup Genotype + Day Postpartum + (1\|Experience) | 7.0 | 6 |
| Pup Genotype + Day Postpartum | 7.1 | 5 |
| null | 9.0 | 3 |
| null +(1\|Experience) | 9.9 | 4 |

Table 6. AICc table for linear mixed models testing the effect of factors on in-cage activity during the dark cycle
