# Supplementary material for: Placental genotype affects early postpartum maternal behaviour
Source: R Soc Open Sci. 2019 Sep 18;6(9):190732. doi: 10.1098/rsos.190732 (PMC6774950; doi:10.1098/rsos.190732)
Supplement: Supplemental File 2 [file rsos190732supp2.rtf]

##litter sizeHB = c(4,3,4,3,2,4,3,3,3,4,4,5,4,4,3,4,6,2,2,2)CS = c(4,4,3,4,6,4,2,3,3,4,4,5,3,5,1,5,6,6,3,4,2,3,3)t.test(HB,CS)##Pregnancy OFTlibrary(nlme)#Line crossesm1 = glm(X.LC~Genotype + DaysP + Genotype:DaysP, data=data, family = “gaussian”)m2 = glm(X.LC~Genotype, data=data, family = “gaussian”)m3 = glm(X.LC~DaysP, data=data, family = “gaussian”)m4 = glm(X.LC~Genotype + DaysP, data=data, family = “gaussian”)m5 = glm(X.LC~1, data=data, family = “gaussian”)library(bbmle)AICctab(m1,m2,m3,m4,m5)#Time in Centerm6 = glm(TIC~ Genotype + DaysP + Genotype*DaysP, data=data, family = “gaussian”)m7 = glm(TIC~Genotype, data=data, family = “gaussian”)m8 = glm(TIC~DaysP, data=data, family = “gaussian”)m9 = glm(TIC~Genotype + DaysP, data=data, family = “gaussian”)m10 = glm(TIC~1, data=data, family = “gaussian”)AICctab(m6,m7,m8,m9,m10)#Latency to enter centerm11 = glm(Latency~Genotype + DaysP + Genotype*DaysP, data=data, family = “gaussian”)m12 = glm(Latency~Genotype, data=data, family = “gaussian”)m13 = glm(Latency~DaysP, data=data, family = “gaussian”)m14 = glm(Latency~Genotype + DaysP, data=data, family = “gaussian”)m15 = glm(Latency~1, data=data, family = “gaussian”)AICctab(m11,m12,m13,m14,m15)#Frozen at the beginning of the trialm16 = glm.nb(Leave~Genotype + DaysP + Genotype*DaysP, data=data)m17 = glm.nb(Leave~Genotype, data=data)m18 = glm.nb(Leave~DaysP, data=data)m19 = glm.nb(Leave~Genotype + DaysP, data=data)m20 = glm.nb(Leave~1, data=data)AICctab(m16,m17,m18,m19,m20)##Pup Retrievallibrary(nlme)HB = factor(Geno, c("HB","WSB"))model1 = lme(Time~ HB + Order + HB*Order, random = ~1|Mom, data = PR, method = "REML")model2 = lme(Time~ HB + Order + HB*Order, random = list(~1|Mom, ~1|Exp), data = PR, method = "REML")anova.lme(model1, model2)anova.lme(model1)library(multcompView)library(lsmeans)marginal = lsmeans(model1,                    ~ HB:Order)cld(marginal,    alpha   = 0.05,     Letters = letters,         adjust  = “bonferroni”)     ##Activity Monitor#light cycle libaray(nlme)model1 = lme(Activity ~ Pup.Genotype, random = ~1|Maternal.ID, data = actl, method = "REML")model2 = lme(Activity ~ Pup.Genotype + Experience, random = ~1|Maternal.ID, data = actl, method = "REML")model3 = lme(Activity ~ Experience, random = ~1|Maternal.ID, data = actl, method = "REML")model4 = lme(Activity ~ 1, random = ~1|Maternal.ID, data = actl, method = "REML")model5 = lme(Activity ~ Pup.Genotype + Day, random = ~1|Maternal.ID, data = actl, method = "REML")model6 = lme(Activity ~ Pup.Genotype + Day + Experience, random = ~1|Maternal.ID, data = actl, method = "REML")model7 = lme(Activity ~ Pup.Genotype + Day + Pup.Genotype*Day, random = ~1|Maternal.ID, data = actl, method = "REML")AICctab(model1, model2, model3, model4, model5, model6, model7,base = T, sort=T,weights=T,delta=T)AICctab(model1, model4, model5, model7,base = T, sort=T,weights=T,delta=T)#dark cyclemodel1 = lme(Activity ~ Pup.Genotype, random = ~1|Maternal.ID, data = actd, method = "REML")model2 = lme(Activity ~ Pup.Genotype + Experience, random = ~1|Maternal.ID, data = actd, method = "REML")model3 = lme(Activity ~ Experience, random = ~1|Maternal.ID, data = actd, method = "REML")model4 = lme(Activity ~ 1, random = ~1|Maternal.ID, data = actd, method = "REML")model5 = lme(Activity ~ Pup.Genotype + Day + Experience, random = ~1|Maternal.ID, data = actd, method = "REML")model6 = lme(Activity ~ Pup.Genotype + Day, random = ~1|Maternal.ID, data = actd, method = "REML")model7 = lme(Activity ~ Pup.Genotype + Day + Pup.Genotype*Day, random = ~1|Maternal.ID, data = actd, method = "REML")AICctab(model1, model2, model3, model4, model5, model6, model7)#ANOVA Dark Cycle 1GroupHB = c(25.75, 12.77083333, 47.27083333, 24.44444444, 21.23611111, 0.055555556, 100.0763889, 0, 21.38194444, 28.75694444, 39.91666667, 36.48611111, 15.03472222)GroupWS = c(16.84027778, 13.29166667, 6.104166667, 18.40277778, 11, 14.31944444, 24.38888889, 15.39583333, 10.36111111, 22.99305556, 10.5862069, 2.652777778, 0, 6.643356643, 22.29166667)Cmbgroup = data.frame(cbind(GroupHB,GroupWS))Stacked_groups2 = stack(Cmbgroup)result2 = aov(values~ind, data = Stacked_groups2)summary(result2)TukeyHSD(result2)#ANOVA Light cycle 4GroupHB = c(2.5, 0.777777778, 3.902777778, 5.3125, 2.069444444, 4.347222222, 3.888888889, 2.527777778, 1.270833333, 0.861111111, 2.423611111, 10.36111111, 4.180555556)GroupWS = c(3.006944444, 4.083333333, 4.520833333, 4.354166667, 4.875, 4.444444444, 12.63888889, 2.6875, 25.45833333, 8.215277778, 2.506944444, 12.40972222, 14.53472222, 4.423611111, 10.19444444)Cmbgroup = data.frame(cbind(GroupHB,GroupWS))Stacked_groups2 = stack(Cmbgroup)result2 = aov(values~ind, data = Stacked_groups2)summary(result2)TukeyHSD(result2)##Suckling/Milk Letdown#Maternal weight changelibrary(nlme)model = lme(Weight.Change~ HB + Time + HB*Time, random = ~1|Mom, data=data, method="REML")model2 = lme(Weight.Change~ HB + Time, random = ~1|Mom, data=data, method="REML")model3 = lme(Weight.Change~ HB, random = ~1|Mom, data=data, method="REML")model4 = lme(Weight.Change~ 1, random = ~1|Mom, data=data, method="REML")library(car)anova(model,model2,model3,model4)anova.lme(model4)#Pup weight changelibrary(nlme)model = lme(WeightChange~ HB + Time + HB*Time, random = ~1|Mom, data=datap, method="REML")model2 = lme(WeightChange~ HB + Time, random = ~1|Mom, data=datap, method="REML")model3 = lme(WeightChange~ HB, random = ~1|Mom, data=datap, method="REML")model4 = lme(WeightChange~ 1, random = ~1|Mom, data=datap, method="REML")library(car)anova(model,model2,model3,model4)anova.lme(model4)#pup weight change at second time pointGroupHpup2 = c(-0.0018, -0.005, 0.001, -0.03, -0.0011, -0.0086, -0.0055, -0.0114, -0.0101, -0.0063, -0.0088, -0.012, -0.0078, -0.0112, -0.0101, -0.009, -0.0095, -0.0092, -0.0068, -0.022,-0.0083,-0.0141,-0.0181, -0.019, -0.0142, -0.0032, -0.004, -0.0042, -0.0049)GroupWpup2 = c(-0.01, 0.031, -0.016, -0.054, -0.01, -0.018, -0.0155, -0.0096, -0.0129, -0.012, -0.012,-0.002, -0.0229, -0.0233, -0.021, -0.016, -0.02, -0.017, -0.023, -0.014, -0.01, 0, -0.01, -0.0164, -0.0199, -0.0134, -0.0125, -0.017, -0.016,-0.014, -0.011, -0.009, -0.019, -0.011, -0.0092, -0.0128, -0.0074)Cmbgroup = data.frame(cbind(GroupHpup2,GroupWpup2))Stacked_groups2 = stack(Cmbgroup)result2 = aov(values~ind, data = Stacked_groups2)summary(result2)TukeyHSD(result2)##USV Production library(nlme)model1 = lme(TOTAL ~ GENOTYPE, random = ~1|LITTER.ID, data = data, method = "REML")model4 = lme(TOTAL ~ 1, random = ~1|LITTER.ID, data = data, method = "REML")AICctab(model1, model4)GroupHB = c(1, 0, 4, 6, 29, 88, 35, 1, 3, 34, 5, 28, 101, 11, 119, 2, 14)GroupWS = c(26, 0, 0, 5, 0, 0, 0, 0, 40, 0, 0)Cmbgroup = data.frame(cbind(GroupHB,GroupWS))Stacked_groups2 = stack(Cmbgroup)result2 = aov(values~ind, data = Stacked_groups2)summary(result2)
